# Cold autoimmune hemolytic anemia resolved by rituximab

**DOI:** 10.4103/0973-6247.67027

**Published:** 2010-07

**Authors:** Massimiliano Palombi, Pasquale Niscola, Alessio Pio Perrotti, Paolo de Fabritiis

**Affiliations:** Department Hematology, S. Eugenio Hospital, Rome, Italy

Sir,

Cold-agglutinin-induced autoimmune hemolytic anemia (AIHA) may be a therapeutic challenge.[1] In this setting, rituximab has been used with favorable results.[2,3] We have recently observed and successfully treated with rituximab a 78-year woman who presented a 2-year history of active cold AIHA unresponsive to prednisone and cyclosporine. The patient reported a long history of hypertension, glaucoma, severe osteoporosis, and osteoarthrosis; she was found to have difficulty walking given the severe damages of ankles and lumbar (L1 to L3) vertebral bodies. At admission, the laboratory evaluation showed a normochromic and normocytic anemia (hemoglobin, Hb = 8.0 g/dL) with reticulocytosis and a typical hemolytic pattern. A comprehensive radiological examination, including a chest X-rays and a total body computed tomography scan, revealed no abnormalities. Gross agglutinants were seen on blood film. An erythroid hyperplasia besides a normal representation of myeloid and megakaryocytic precursors resulted from the examination of the bone marrow smears and by a trephine biopsy. Immunohaematological investigations revealed a strongly positive direct antiglobulin test (DAT) and anti-C3 antisera with the presence of a cold IgG (1:512) active from 3°C to 20°C. Therefore, a salvage therapy with rituximab as a 4-h intravenous infusion at the dose of 375 mg/m^2^ once weekly for a total of four doses was given. No adverse reactions were observed. To begin after the second dose of rituximab, Hb levels constantly increased and this finding was paralleled by the decrease of the reticulocyte count. Three months after the last dose of rituximab, the DAT became negative. To date, at the 13-months follow-up, the patient’s Hb level and serum markers of hemolysis are normal and the DAT is negative. AIHA due to cold agglutinins is a difficult to treat disease presenting a variable response to corticosteroids, immunosuppressive drugs, alkylating cytostatics, interferon, corticosteroids, and splenectomy.[1] Our patient lost the therapeutic response to prednisone and cyclosporine which had been administered for many years. The present report adds another case to the relatively sparse data on idiopathic cold AIHA treated with rituximab, for which favorable results have been reported[2,3] with a response rate of approximately 60% and an acceptable cost-effective profile.[4] In conclusion, in our experience, rituximab was safely administered and provided the means to obtain sustained remissions, representing an optimal tool in this setting even in order to spare bone loss, skeletal complications, and other steroid-related side effects, which may be particularly devastating and aggravating the frailty of the older patients.
